# Vibration State Identification of Hydraulic Units Based on Improved Artificial Rabbits Optimization Algorithm

**DOI:** 10.3390/biomimetics8020243

**Published:** 2023-06-08

**Authors:** Qingjiao Cao, Liying Wang, Weiguo Zhao, Zhouxiang Yuan, Anran Liu, Yanfeng Gao, Runfeng Ye

**Affiliations:** 1 School of Water Conservancy and Hydropower, Hebei University of Engineering, Handan 056038, China; caoqingjiao@hebeu.edu.cn (Q.C.); gaoyanfeng@hebeu.edu.cn (Y.G.); 2 Hebei Key Laboratory of Intelligent Water Conservancy, Hebei University of Engineering, Handan 056038, China; 3 Hebei Zhanghewan Pumped Storage Power Generation Co., Ltd., Shijiazhuang 050300, China; yuan94218@163.com; 4 School of Water Conservancy, North China University of Water Resources and Electric Power, Zhengzhou 450045, China; liu_anran@163.com; 5 Graduate School, Hebei University of Engineering, Handan 056038, China

**Keywords:** variational mode decomposition, artificial rabbits optimization, support vector machine, vibration state of hydraulic units, signal identification

## Abstract

To improve the identification accuracy of the vibration states of hydraulic units, an improved artificial rabbits optimization algorithm (IARO) adopting an adaptive weight adjustment strategy is developed for optimizing the support vector machine (SVM) to obtain an identification model, and the vibration signals with different states are classified and identified. The variational mode decomposition (VMD) method is used to decompose the vibration signals, and the multi-dimensional time-domain feature vectors of the signals are extracted. The IARO algorithm is used to optimize the parameters of the SVM multi-classifier. The multi-dimensional time-domain feature vectors are input into the IARO-SVM model to realize the classification and identification of vibration signal states, and the results are compared with those of the ARO-SVM model, ASO-SVM model, PSO-SVM model and WOA-SVM model. The comparative results show that the average identification accuracy of the IARO-SVM model is higher at 97.78% than its competitors, which is 3.34% higher than the closest ARO-SVM model. Therefore, the IARO-SVM model has higher identification accuracy and better stability, and can accurately identify the vibration states of hydraulic units. The research can provide a theoretical basis for the vibration identification of hydraulic units.

## 1. Introduction

With the continuous development of the new energy industry, hydropower, a type of renewable and clean energy, has received more and more attention. As the core equipment of hydroelectric power generation, the operating states of hydraulic units are related to power generation efficiency, economic benefits and production safety [1]. Vibration is an important factor that affects the stable operation of hydraulic units. Comprehensive, effective and accurate classification and identification of vibration states are the premise of monitoring the operating states of hydraulic units, and it is very necessary to improve the identification accuracy of the vibration states of hydraulic units to judge the stable operation of hydraulic units.

Since the hydraulic unit is subjected to the combined action of mechanical, hydraulic and electrical factors, its vibration signal shows obvious non-stationary and non-linear characteristics [2]. For the identification of the vibration states of hydraulic units, processing and extracting the feature vector of the vibration state signals is an indispensable part. Seyrek et al. [3] proposed three decomposition methods, namely, empirical mode decomposition (EMD), ensemble empirical mode decomposition (EEMD), and variational mode decomposition (VMD), which are used to decompose and identify chatter frequency bands. Joshuva et al. [4] identified the states of a wind turbine blade based on its vibration pattern and used VMD for signal pre-processing. Wang et al. [5] proposed a multi-objective particle swarm optimization (MOPSO) algorithm to optimize the VMD parameters, and it is applied to the composite fault diagnosis of the gearbox. Zhang et al. [6] proposed an adaptive VMD method based on the grasshopper optimization algorithm (GOA) to analyze the vibration signals of rotating machinery. Qaisar et al. [7] proposed a new method to identify arrhythmias by processing electrocardiogram signals. The solution is based on an effective hybridization of the multi-rate processing, QRS selection, variational mode decomposition, feature extraction from modes, metaheuristic optimization-based feature selection, and machine learning algorithms. Mazzeo et al. [8] proposed an effective computational strategy for bridge modal identification based on its free vibration response. To make the bearing run under variable working conditions, Liu et al. [9] proposed a feature extraction method based on SHO-VMD decomposition and multi-feature parameter fusion. Ni et al. [10] proposed a fault information-guided VMD (FIVMD) method to extract repetitive transient signals from weak bearings.

In recent years, many scholars have proposed different methods in signal feature vector identification combined with optimization algorithms. Hatiegan et al. [11] measured and analyzed the vibration data of the hydraulic turbine under different working conditions during operation. Jose et al. [12] proposed a practical method for the early detection of internal leakage faults in boom actuators of mobile hydraulic machines. This method used pressure and boom angle displacement signals to train and verify the SVM classifier, and used the binary version of particle swarm optimization (PSO) for feature selection. Jena et al. [13] used the unsubtracted wavelet transform (UWT) to denoise the signal and identified and located the gear defects in the time domain of the vibration signal. Alsaiari et al. [14] used artificial rabbits optimization (ARO) to establish a multi-layer perception (MLP) [15] coupling model to predict the water yield of solar stills (SSs) with different designs. To improve the load frequency control (LFC) of IMG, Khalil et al. [16] applied ARO to IMG to simultaneously adjust the controller parameters of multiple controlled sources. Pei et al. [17] used the SVM improved by GA and the shuffled frog leaping algorithm (SFLA) [18] to identify fault features, respectively. Ma et al. [19] proposed an identification method based on singular value decomposition (SVD) and MPSO-SVM. Wang et al. [20] used VMD to decompose the acoustic vibration signal of water pipes, extracted three feature vectors, formed a new feature vector through multi-source information fusion, and finally input it into the SVM classifier for leak identification. Li et al. [21] used manta ray foraging optimization (MRFO) [22] to optimize the parameters of the proposed SVM for short-term load forecasting. Zhang et al. [23] proposed an optical fiber vibration signal identification method based on HOSA-SVM and EMD-AWPP. Saari et al. [24] trained the model by using the fault features extracted from the vibration signals as the input of one-class SVM and realized the automatic detection and identification of wind turbine bearing faults. Ruan et al. [25] use the phase space reconstruction (PSR) method to extract the feature set that represents the health condition of the trip mechanism from the vibration signal and input it into the fault identification model based on SVM.

The ARO has the advantages of simple operation, few parameters to be set and strong optimization performance, but its convergence accuracy is low, the convergence speed is slow and it is easy to fall into local optimization. Therefore, the adaptive weight adjustment method is selected to improve the ARO algorithm. The selection of penalty parameters and kernel function parameters of the SVM is the key to affecting signal identification [17]. This paper proposes an identification method of the ARO algorithm with the adaptive weight adjustment strategy to optimize SVM. The variational mode decomposition (VMD) has high computing efficiency, a low number of decomposition layers, no mode aliasing and endpoint effects [26], and its intrinsic mode component (IMF) contains most of the effective information. Therefore, the VMD method is used to decompose the different states of the vibration signals of the hydraulic units, select the IMF component, and extract the time-domain feature indicator of the IMF component. Through multi-source information fusion, multiple time-domain feature vectors are combined into a new multi-dimensional feature vector and finally input into the IARO-SVM model for vibration state identification. The IARO-SVM model is compared with the ARO-SVM model, the ASO-SVM model, the PSO-SVM model and the WOA-SVM model. The results show that the IARO-SVM model has a higher identification accuracy and can effectively distinguish different signal states of vibration states.

The main motivation of this paper is summarized as follows.

- The stable operation of hydraulic units is very important for the safe production of hydropower stations. Vibration state monitoring is the premise of analyzing whether the hydraulic unit is operating stably. It is very necessary to improve the identification accuracy of the vibration states of hydraulic units to guarantee the secure operation of the hydraulic units;
- Optimization algorithms are widely used in signal classification and identification. However, many optimization algorithms have some shortcomings. Therefore, according to the shortcomings of the ARO algorithm, this paper proposes an improvement strategy based on the adaptive adjustment of the weight to improve the identification accuracy of the vibration states of the hydraulic units;

The main contributions of this study are highlighted as follows.

- A method of adaptively changing the inertia weight according to the current rabbit population distribution is proposed, which is used to improve the optimization ability of the ARO algorithm;
- A total of 23 benchmark functions are used to test the performance of the IARO algorithm. The experimental results show that the IARO algorithm has good exploration and exploitation abilities;
- Through the application of vibration state signals of the hydraulic units, the identification method of SVM optimized by the IARO algorithm is verified. The experimental results show that the IARO-SVM model can well identify the different vibration states of hydraulic units, and provide a guarantee for the stable operation of hydraulic units.

The residual section of this paper is as follows. Section 2 describes the basic principles of the VMD algorithm, the ARO algorithm and SVM. In Section 3, a method of adaptively changing the inertia weight according to the current rabbit population distribution is proposed to improve the original version. Section 4 describes testing the performance of the IARO algorithm using 23 benchmark functions. Section 5 verifies the practicality of the IARO-SVM model in engineering practice by identifying the vibration states of hydraulic units. Section 6 introduces the conclusion and future development direction.

## 2. The Proposed Method

### 2.1. Variational Mode Decomposition Algorithm

To solve the problems of modal aliasing and endpoint effects in signal decomposition by classical recursive decomposition algorithms such as local mean decomposition (LMD) and EMD, Dragomiretskiy et al. [27] proposed a completely non-recursive VMD algorithm. The VMD [28,29] has strong noise robustness, by which non-stationary sequences with high complexity and strong nonlinearity can be decomposed into several relatively stationary subsequences with different frequency scales [30]. Its core is the construction and solution of variational problems, which describe the VMD variational problem as follows: assume that the original signal $y\left(t\right)$ consists of K eigenmode functions $\left\{u_{k}\left(t\right)\right\},\left(k=1,2,\cdots K\right)$ with different center frequencies and limited bandwidth, and constrain the sum $\sum_{k=1}^{K}u_{k}\left(t\right)$ of each eigenmode function to restore the original signal $y\left(t\right)$. The constrained variational problem is expressed as follows:

(1) $$\left\{\begin{array}{l} \underset{\left\{u_{k}\right\},\left\{\omega_{k}\right\}}{\min}\left({\sum_{k}\left\|\partial_{t}\left[\left(\delta (t)+\frac{j}{\pi t}\right)⊗u_{k}(t)\right]\right.\left.e^{-j\omega_{k}t}\right\|}^{2}\right)\\ s.t.\sum_{k}u_{k}\left(t\right)=y\left(t\right) \end{array}\right.$$

where δ(t) is the pulse function, j represents imaginary number; ⊗ represents convolution, $y\left(t\right)$ is the original signal, $\omega_{k}$ is the center frequency of each modal component $u_{k}(t)$, and $\partial_{t}$ represents the derivation of time.

To solve the above variational model Equation (1), the quadratic penalty factor and the Lagrange multiplier are introduced to transform the above constrained variational problem into an unconstrained variational problem:

(2) $$\begin{array}{l} L\left(\left\{u_{k}\right\},\left\{\omega_{k}\right\},\lambda \right)=\alpha \sum_{k=1}^{K}\left\|\partial_{t}\left[\left(\delta (t)+\frac{j}{\pi t}\right)⊗u_{k}(t)\right]\right.{\left.e^{-j\omega_{k}t}\right\|}_{2}^{2}\\ +\left\|y(t)-\sum_{k=1}^{K}u_{k}(t)\left\|{}_{2}^{2}+\langle \lambda (t),y(t)-\sum_{k=1}^{K}u_{k}\left(t\right)\rangle \right.\right. \end{array}$$

where $\langle \cdot \rangle$ represents the inner product, ${\left\|y(t)-\sum_{k=1}^{K}\left.u_{k}(t)\right\|\right.}_{2}^{2}$ is the second penalty, α reduce the influence of Gaussian noise on signal reconstruction, and λ(t) ensure the strictness of the constraints.

On the basis of the transformation variational problem, the alternating direction method of multipliers (ADMM) is used to alternately update $u_{k}^{n+1}$, $\omega_{k}^{n+1}$, and $\lambda^{n+1}$ to solve the ‘saddle point’ of Equation (2), that is, to solve the optimal solution of the variational problem. The iteration process is stopped when the following conditions are met:

(3) $${{\sum_{k}\left\|{\overset{⌢}{u}}_{k}^{n+1}-\left.{\overset{⌢}{u}}_{k}^{n}\right\|\right.}_{2}^{2}/\left\|\left.{\overset{⌢}{u}}_{k}^{n}\right\|\right.}_{2}^{2}<\varepsilon$$

where ε is the discrimination accuracy.

After the end of the cycle, K modal components $u_{k}(t)$ can be obtained by inverse Fourier transform of ${\overset{⌢}{u}}_{k}\left(\omega \right)$.

### 2.2. Artificial Rabbits Optimization Algorithm

**Figure 1:**
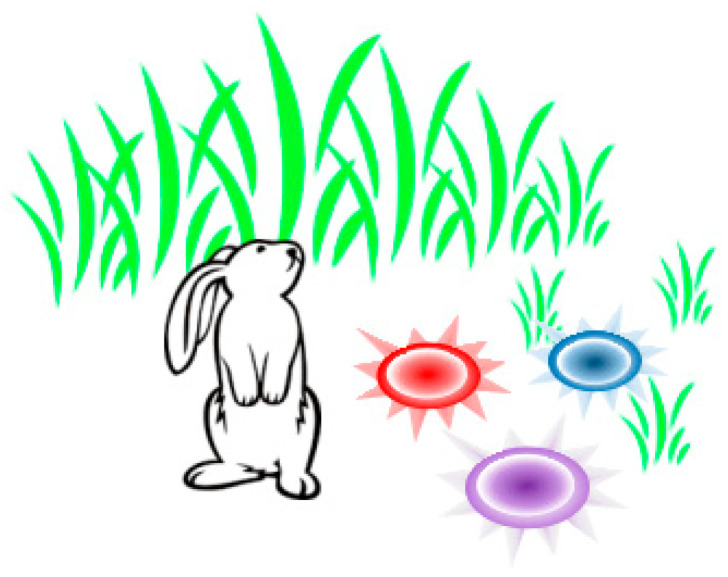
Illustrates that a cunning rabbit has three burrows [31].

The ARO is a new bionic heuristic algorithm [31] inspired by the famous Chinese proverbs “Rabbits don’t eat the grass on the edge of the nest” and “A cunning rabbit has three burrows”. Figure 1 depicts the three burrows of a cunning rabbit. The ARO algorithm adopts the detour foraging and hiding strategies.

#### 2.2.1. Detour Foraging (Exploration)

Assume that each rabbit in the population has its own area, each area has edible grass and d burrows, and rabbits always visit the foraging position randomly. The mathematical model of the detour foraging is as follows [31]:

(4) $$v_{i}(t+1)=x_{j}(t)+R•(x_{i}(t)-x_{j}(t))+round(0.5•(0.05+r_{1}))•n_{\begin{array}{l} 1 \end{array}},i=1,\cdots ,n,j\neq i$$

(5) R=L•C

(6) $$L=(e-e^{{\left(\frac{t-1}{T}\right)}^{2}})•\sin(2\pi r_{2})$$

(7) $$c(k)=\left\{\begin{array}{l} 1\\ 0 \end{array}\right.\begin{array}{l} if\begin{array}{cc} k==g(l) & \end{array}\\ \begin{array}{l} \begin{array}{cc} & else \end{array} \end{array} \end{array}k=1,\cdots ,d\begin{array}{cc} and & l \end{array}=1,\cdots ,\left\lceil r_{3}•d\right\rceil$$

(8) g=randperm(d)

(9) $$n_{1}∼N(0,1)$$

where $v_{i}(t+1)$ is the candidate position of the ith rabbit at the time t+1, $x_{i}(t)$ is the position of the ith rabbit at the time t, n is the number of rabbits, d is the dimension, T is the maximum number of iterations, $\left\lceil •\right\rceil$ is the ceiling function. L is the step size. $n_{1}$ is subject to the standard normal distribution. c is the mapping vector. 

#### 2.2.2. Random Hiding (Exploitation)

To reduce the probability of being hunted, a rabbit randomly chooses a burrow to hide. The ith rabbit is in the ith burrow, and the mathematical expression is as follows [31]:

(10) $$b_{i,j}(t)=x_{i}(t)+H\cdot g\cdot x_{i}(t),i=1,\cdots ,n\;\mathrm{and}\;j=1,\cdots ,d$$

(11) $$H=\frac{T-t+1}{T}\cdot r_{4}$$

(12) $$n_{2}∼N(0,1)$$

(13) $$g(k)=\left\{\begin{array}{l} 1\;if\;k==j\\ 0\;else \end{array}\right.k=1,\cdots ,d$$

To simulate this random hiding behavior of rabbits, the following mathematical equations are given [31]:

(14) $$v_{i}(t+1)=x_{i}(t)+R\cdot (r_{4}\cdot b_{i,r}(t)-x_{i}(t)),i=1,\cdots ,n$$

(15) $$g_{r}(k)=\left\{\begin{array}{l} 1\;if\;k==\left\lceil r_{5}\cdot d\right\rceil \\ 0\;else \end{array}\right.k=1,\cdots ,d$$

(16) $$b_{i,r}(t)=x_{i}(t)+H\cdot g_{r}\cdot x_{i}(t)$$

where $b_{i,r}$ is a randomly selected burrow from d burrows. $r_{4}$ and $r_{5}$ are two random numbers between (0,1). According to Equation (14), the ith rabbit randomly selects a burrow from its d burrows to update its position. 

After the detour foraging and random hiding are completed, the position of the ith rabbit is updated as follows [31]:

(17) $$x_{i}(t+1)=\left\{\begin{array}{ll} x_{i}(t) & f(x_{i}(t))\leq f(v_{i}(t+1))\\ v_{i}\left(t+1\right) & f(x_{i}(t))>f(v_{i}(t+1)) \end{array}\right.$$

#### 2.2.3. Energy Shrink (Switch from Exploration to Exploitation)

An energy factor needs to be designed to simulate the transition from exploration to exploitation throughout the iteration. The energy factor is defined as follows [31]:

(18) $$A(t)=4(1-\frac{t}{T})\ln\frac{1}{r}.$$

**Figure 2:**
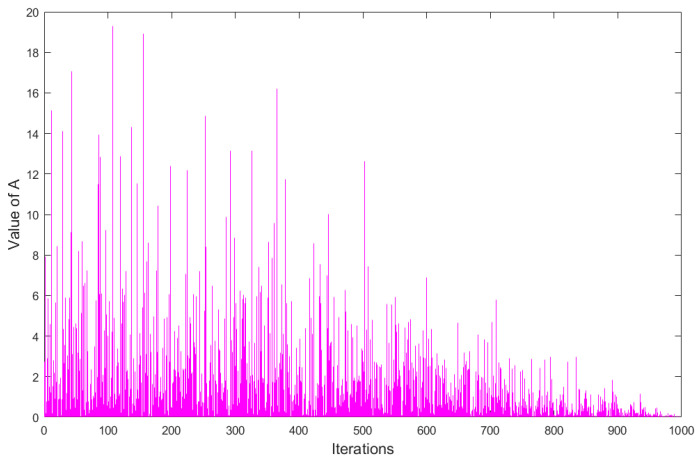
The behavior of A in 1000 iterations.

The behavior of A over 1000 iterations is depicted in Figure 2. In Figure 2, when the energy factor $A\left(t\right)>1$, rabbits tend to randomly explore the areas of different rabbits for foraging in the exploration phase; thus, detour foraging occurs. When the energy factor $A\left(t\right)\leq 1$, rabbits tend to randomly exploit their own burrows in the exploitation phase; thus, random hiding occurs. 

### 2.3. Multi-Classification Design of SVM

SVM is a class of generalized linear classifiers for binary classification of data according to supervised learning, and its decision boundary is the maximum margin hyperplane for solving learning samples [32,33].

Given input data and learning objectives $X=\left\{X_{1},\cdots ,X_{N}\right\}$, $y=\left\{y_{1},\cdots ,y_{N}\right\}\left(X_{i}\in R^{n};y_{i}\in \left\{-1,+1\right\}\right)$. If the samples are linearly separable, SVM transforms the classification problem into a convex quadratic optimization problem.

(19) $$\left\{\begin{array}{l} \min\frac{1}{2}{\left\|\omega \right\|}^{2}+C\sum_{i=1}^{n}\xi_{i}\\ s.t.\begin{array}{cc} & y_{i}\left(\omega \cdot X_{i}+b\right)\geq 1-\xi_{i} \end{array} \end{array}\right.$$

where ω is the weight. C is the penalty factor. ξ is the relaxation factor. b is the bias constant.

The optimal classification decision function obtained is

(20) $$sign\left[y_{i}\left(\omega \cdot X_{i}+b\right)\right].$$

When the order of the polynomial kernel is not 1, a SVM can be obtained.

The SVM optimization problem is

(21) $$\left\{\begin{array}{l} \min\frac{1}{2}{\left\|\omega \right\|}^{2}+C\sum_{i=1}^{n}\xi_{i}\\ s.t.\begin{array}{cc} & y_{i}\left[\omega \cdot \phi \left(X_{i}\right)+b\right]\geq 1-\xi_{i}\begin{array}{cc} & \xi_{i}\geq 0 \end{array} \end{array} \end{array}\right.$$

The optimal classification decision function obtained is

(22) $$sign\left[y_{i}\left(\omega \cdot \phi \left(X_{i}\right)+b\right)\right].$$

The binary classification SVM can be extended to multi-classification identification based on a multi-classification strategy. The “one-to-one” (OVO) strategy is combined with SVM to form a multi-classifier, which can obtain better classification performance [34]. When solving the multi-classification problem of K-class signals, constructing a two-class SVM sub-classifier between any two categories of samples, a total of $K\left(K-1\right)/2$ sub-classifiers need to be constructed. In this study, since the Gaussian radial basis function (RBF) has fewer parameters, it is used as the kernel function of the SVM [17].

## 3. Improvement of Artificial Rabbit Optimization Algorithm

The ARO has the advantages of simple operation, few parameters to be set and strong optimization performance, but there are also points that can be improved, such as convergence accuracy, convergence speed, and local optima avoidance. Using dynamic inertial weights, in the early stage of algorithm iteration, due to the relatively dispersed population, larger weight values can be assigned to accelerate the global search ability of the algorithm. In the later stage of iteration, the algorithm can adaptively change the size of the weight value according to the distribution of individuals in the current population, so that it can search finely around the optimal solution and accelerate the convergence speed.

According to the inspiration of Kong et al. [35] on the improvement of the whale optimization algorithm (WOA), this paper proposes a method to improve the ARO algorithm, namely, the ARO with adaptive weight (IARO).

### 3.1. Adaptive Inertia Weight

The inertia weight is an important parameter in improving the ARO algorithm. The appropriate weight value can improve the optimization ability of the algorithm. However, the improper selection of linear inertia weight adjustment strategies will affect the convergence speed of the algorithm. Therefore, this paper proposes a method to adaptively change the weight value according to the current rabbit population distribution, which is as follows:

(23) $$w=a_{1}\cdot \left(P_{worst}^{j}-P_{best}^{j}\right)+\frac{a_{2}}{t}\cdot \left(x_{i}^{\max j}-x_{i}^{\min j}\right)$$

(24) $$x_{i}^{\max\begin{array}{l} j \end{array}}=\underset{j\in \left[1,d\right]}{\max\begin{array}{l} x_{i}^{j} \end{array}}\begin{array}{ll} & i=1,\cdots ,n \end{array}$$

(25) $$x_{i}^{\max\begin{array}{l} j \end{array}}=\underset{j\in \left[1,d\right]}{\min\begin{array}{l} x_{i}^{j} \end{array}}\begin{array}{ll} & i=1,\cdots ,n \end{array}$$

(26) $$a_{1}=\cos(0.5\pi \cdot r)$$

(27) $$a_{2}=1-a_{1}$$

where t represents the number of iterations of the current population. $P_{iworst}$ and $P_{ibest}$ are the position vector of the worst rabbit and the position vector of the optimal rabbit in the current rabbit population, respectively. The obvious difference between the proposed inertia weight and the weight in [35] is that our method uses the current population information to update the lower and upper boundaries to adaptively adjust the search space; another difference between them is that our method uses two random coefficients to compromise the maximum individual distance and the distance in each dimension. The adaptive adjustment for the weight of the current rabbit in the random hidden update position is as follows:

(28) $$v_{i}(t+1)=w\cdot x_{i}(t)+R\cdot (r_{4}\cdot b_{i,r}(t)-x_{i}(t))$$

After introducing the adaptive adjustment weight strategy, the algorithm can adaptively change the weight size according to the distribution of the current rabbit population. In the early stage of algorithm iteration, if the rabbit population falls into the local optimal solution, and there is little difference between the optimal solution and the worst solution, the value of $a_{1}\cdot \left(P_{worst}^{j}-P_{best}^{j}\right)$ is not affected by the population distribution, and this term can still get a larger weight value of w, so as to avoid falling into a small search range in the initial iteration. As the number of iterations increases, the value of $a_{1}\cdot \left(P_{worst}^{j}-P_{best}^{j}\right)$ will gradually become smaller, and its influence on the weight w will decrease. If the algorithm does not get the optimal solution at this time, the design of $a_{2}\cdot \left(x_{i}^{\max j}-x_{i}^{\min j}\right)/t$ can play a leading role in the weight value w, which can make the algorithm search with a larger step size. The advantage of this adaptive weight adjustment method is determined by two parts. The first part changes the population when the number of iterations is too large, and the second part changes the population when it falls into the local optimal.

### 3.2. The Specific Procedure of the IARO Algorithm

**Figure 3:**
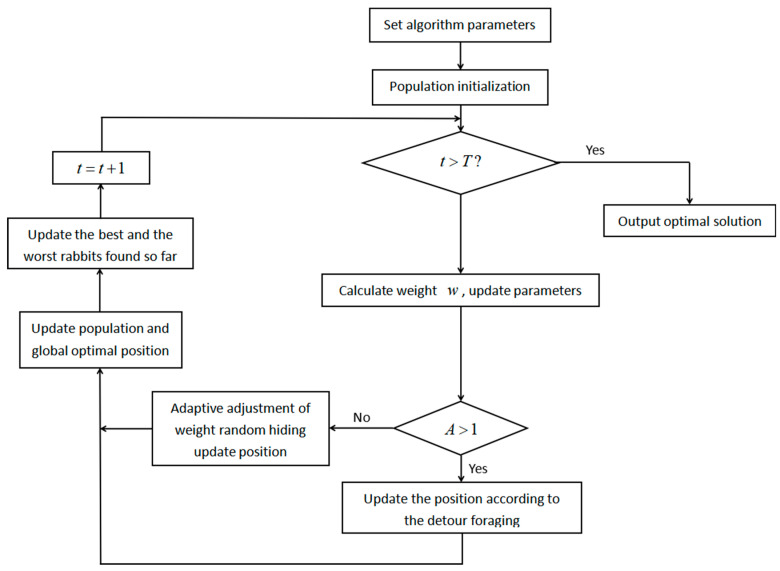
Flow chart of the IARO algorithm.

The flow chart of the IARO is shown in Figure 3, with the steps as follows: 

Step 1: Parameter initialization. Set the population size N, the maximum iteration number T, and the parameter $a_{1},a_{2}$;

Step 2: Population initialization. The initial solution is randomly generated, the individual fitness degree $\left\{F\left(X_{i}\right),i=1,2,\cdots ,N\right\}$ is calculated, and the best and worst rabbits found so far are recorded;

Step 3: Calculate the weight w according to Equation (23); update parameters L, c, R, A, H, g and b;

Step 4: If A>1, update the current rabbit position according to Equation (4);

Step 5: If A≤1, update the current rabbit position according to Equation (28);

Step 6: The population is updated and the best and worst rabbits found so far are updated;

Step 7: If the algorithm reaches the maximum number of iterations, output the optimal solution; otherwise, return to Step 3.

### 3.3. Energy Shrink of the IARO Algorithm

**Figure 4:**
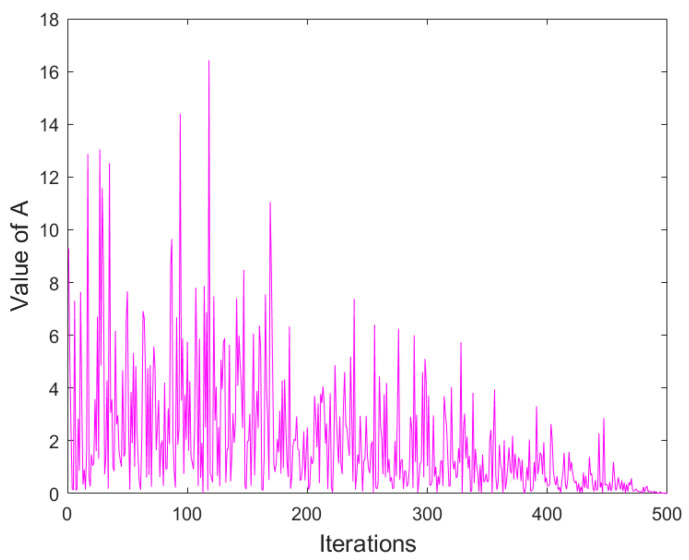
Energy shrink of the IARO algorithm.

In the iterative process of the IARO algorithm, the transition process of the energy factor from exploration to exploitation is shown in Figure 4.

## 4. Experimental Results and Analysis

To test the performance of the IARO algorithm, 5 algorithms, including IARO, ARO, PSO, ASO (atom search optimization) [36] and WOA, are compared, and 23 benchmark functions are used in the experiment [37]. The 23 benchmark functions are shown in Table 1, Table 2 and Table 3 including 7 unimodal functions, 6 multimodal functions and 10 low-dimensional multimodal functions. The IARO algorithm has been carried out using the MATLAB 9.7 (R2019b) desktop computer running Windows 10 64-bit with an Intel(R) Core(TM) i5-9400F CPU 2.9 GHz processor and 8.00 GB RAM. The initial population size of all algorithms is set to 50, the maximum number of iterations is 300, and each optimization algorithm runs repeatedly 30 times. The performance of the algorithm is analyzed and evaluated by four indicators such as the mean value (Mean), standard deviation (Std), worst value (Worst) and best value (Best) of the optimal solution found so far.

### 4.1. Exploitation Assessment

**Figure 5:**
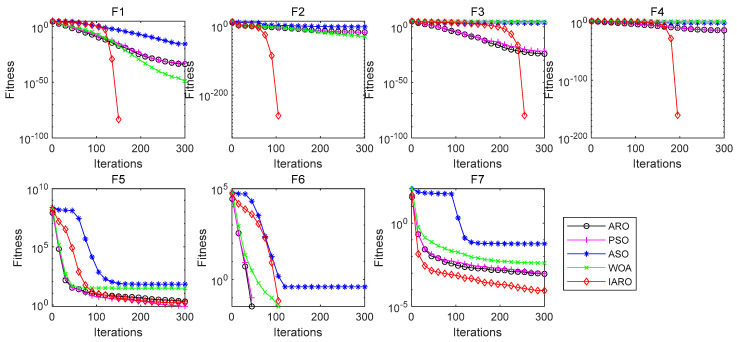
Convergence curve of the unimodal functions.

Since the unimodal function (F1–F7) has only one optimal value, it can be used to test the exploitation of the algorithm. The results of each indicator of the six algorithms in different unimodal test functions are shown in Table 4. The best value of each indicator has been marked in bold. Figure 5 shows the convergence curves of the five algorithms in each unimodal test function.

From Table 4, for functions F1, F2, F3, F4 and F7, the ‘Mean’, ‘Std’, ‘Worst’ and ‘Best’ of the IARO algorithm are the best among the five algorithms. For function F6, the four indicators of the IARO and ARO, WOA and PSO algorithms are the best. For function F5, the ‘Std’ provided by the WOA algorithm is the best. The ‘Mean’, ‘Worst’ and ‘Best’ provided by the PSO algorithm are the best. Therefore, for the unimodal functions, the IARO algorithm is superior to other algorithms when dealing with the unimodal function.

From Figure 5, for functions F1, F2, F3, F4 and F7, the IARO algorithm has higher convergence accuracy and faster convergence speed. For function F6, the convergence accuracy of the IARO and ARO, WOA, and PSO algorithms is the best, but the convergence speed of the IARO algorithm is slower than the other three algorithms. For function F5, the convergence accuracy and convergence speed of the IARO algorithm are lower than that of the PSO algorithm but higher than other algorithms. Therefore, for functions F1, F2, F3, F4, F6 and F7, the convergence accuracy of the IARO algorithm is optimal. For function F5, the convergence accuracy of the IARO algorithm is only lower than that of the PSO algorithm. For functions F1, F2, F3, F4 and F7, the convergence speed of the IARO algorithm is the fastest. For functions F5 and F6, the convergence speed of the IARO algorithm is in the middle of the five algorithms. From the above analysis, the IARO algorithm performs the best in Figure 5.

### 4.2. Exploration Assessment

**Figure 6:**
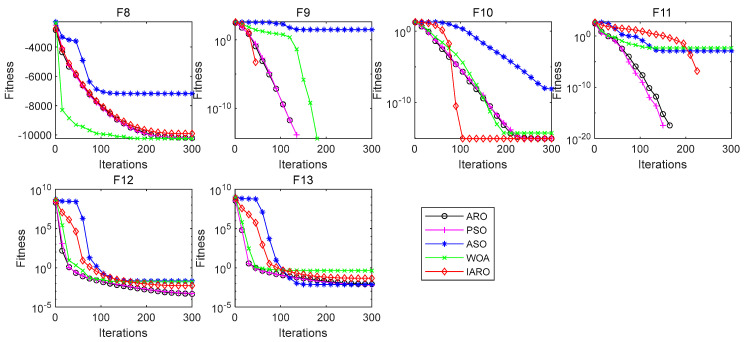
Convergence curve of algorithms on the multimodal functions.

**Figure 7:**
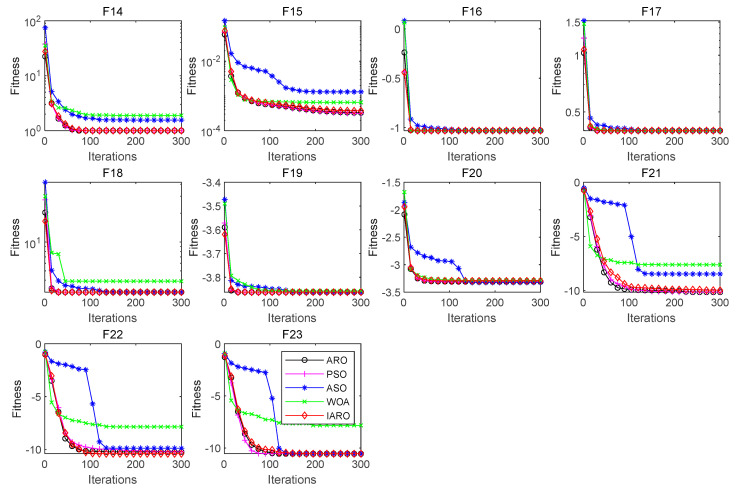
Convergence curve of the fixed dimension multimodal test functions.

Since the multimodal function (F8–F23) has multiple extreme values, it can be used to test the exploration ability of the algorithm. The results for each indicator of the five algorithms on different multimodal functions are shown in Table 5 and Table 6. Figure 6 and Figure 7 show the convergence curves of the five algorithms in each multimodal function. 

From Table 5, for function F8, the ‘Std’ and ‘Worst’ of the IARO algorithm are the best among the five algorithms. The ‘Mean’ and ‘Best’ of the ARO and WOA, PSO algorithms are the best among the five algorithms. For function F9, the four indicators of the IARO and ARO, WOA, and PSO algorithms are the best. For function F10, the four indicators of the IARO and ARO algorithms are the best. For function F11, the four indicators of the IARO and ARO, PSO algorithms are the best. For function F12, the ‘Mean’ and ‘Best’ of the ARO algorithm are the best. Therefore, for the multimodal functions, the IARO shows better exploration when tackling multimodal functions.

From Figure 6, for function F5, the IARO algorithm has a faster convergence speed. For function F10, the IARO algorithm has higher convergence accuracy and faster convergence speed. For functions F8, F12 and F13, the convergence accuracy and convergence speed of the IARO algorithm are at the middle level among the five algorithms.

From Table 6, for functions F8 and F15, the four indicators of the IARO and ARO algorithms are the best among the five algorithms. For functions F16 and F17, the ‘Mean’, ‘Worst’ and ‘Best’ of the five algorithms are the best. The ‘Std’ of the IARO algorithm is the best. For function F18, the ‘Mean’, ‘Worst’ and ‘Best’ of the IARO and ARO, PSO, and ASO algorithms are the best. The ‘Std’ of the ARO algorithm is the best. For function F19, the ‘Mean’, ‘Worst’ and ‘Best’ of the IARO and ARO, PSO, and ASO algorithms are the best. For function F22, the four indicators of the IARO algorithm are the best. For function, F23, the ‘Mean’, ‘Worst’ and ‘Best’ of the IARO and ARO, PSO, and ASO algorithms are the best.

From Figure 7, for functions F14, F15, F16, F17, F18, F19 and F20, the IARO algorithm has higher convergence accuracy and faster convergence speed. For functions F21, F22 and F23, the IARO algorithm has higher convergence accuracy, and the convergence speed is significantly higher than that of the ASO and WOA algorithms.

By using 23 benchmark functions to test the performance of five algorithms, the minimum values and convergence curves of four indicators of each algorithm in different benchmark functions are obtained. Through comparative analysis, it can be concluded that the IARO algorithm has the strongest ability to find the optimal solution.

## 5. IARO for Vibration State Identification of Hydraulic Units

### 5.1. Acquisition of Experimental Data

The Zhanghewan Pumped Storage Power Station is located in Shijiazhuang City, Hebei Province, with a total installed capacity of 1000 MW. Four single-stage mixed-flow reversible pump turbine units with a single capacity of 250 MW are installed. In the power station, the rated head of the water pump turbine unit is 305 m, the rated speed is 333.3 r/min, the annual power generation is 1.675 billion kW·h, the annual pumping power is 2.204 billion kW·h, the annual power generation utilization time is 1675 h, the annual pumping utilization time is 2204 h, and the comprehensive efficiency coefficient of the power station is 0.76. After the completion of the power station, it is connected to the power grid in southern Hebei and undertakes the tasks of peak shaving, valley filling, frequency modulation, phase modulation and emergency backup in the system [38,39].

The experimental data comes from the 1# Unit data of the Zhanghewan Pumped Storage Power Station. The selected three vibration states are the stability test of power generation condition, the Karman vortex street phenomenon test and the simulated fault shutdown test. For each vibration state, three parts are selected as the sensor measuring points, and the X direction and the Y direction are arranged in coordination. The six measuring points are, respectively, the X and Y directions of the upper guide bearing, the X and Y directions of the water guide bearing and the X and Y directions of the lower guide bearing.

The experimental data of three vibration states are classified as shown in Table 7. Each vibration state is classified, that is, the power generation condition stability test No. 1, Karman vortex street phenomenon test No. 2 and simulated fault shutdown test No. 3.

### 5.2. Vibration Signal Processing and Extraction

The different vibration states of hydraulic units are studied experimentally, and the vibration signals are denoised and extracted by the VMD decomposition. Firstly, the center frequency method [40] is used to determine the number of decomposition layers K. Secondly, the residual index method [41] is used to determine the update step tau. In each vibration state, the number of decomposition layers K and the update step tau of the six measuring points are shown in Table 8. Finally, according to the data in Table 8, for each vibration state, the vibration signals of six measuring points are decomposed by VMD, respectively, and the corresponding IMF components can be obtained.

In order to unify the number of IMF components, according to Table 8, K=10 is selected as the number of decomposition modes extracted from each group of vibration signals; that is, IMF1-IMF10 is selected as the decomposition mode $X=[X_{1},X_{2},X_{3},X_{4},X_{5},X_{6},X_{7},X_{8},X_{9},X_{10}]$.

### 5.3. Feature Vectors Selection

**Figure 8:**
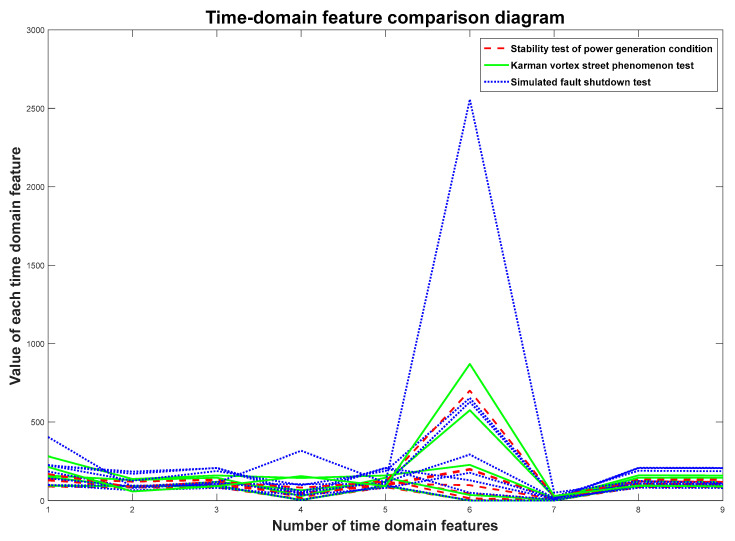
Time-domain feature comparison chart.

For each vibration state, the signals of six measuring points are averaged. The obtained mean value is substituted into the time-domain feature model. After calculation and analysis, nine time-domain features are selected as the feature vectors $Y=[Y_{1},Y_{2},Y_{3},Y_{4},Y_{5},Y_{6},Y_{7},Y_{8},Y_{9}]$ of each vibration state, which are the maximum value, minimum value, mean value, peak-to-peak value, rectification mean value, variance, standard deviation, root mean square and square root amplitude. The comparison of the nine time-domain features of the three vibration states is shown in Figure 8.

### 5.4. SVM Classification and Identification Model Construction

**Figure 9:**
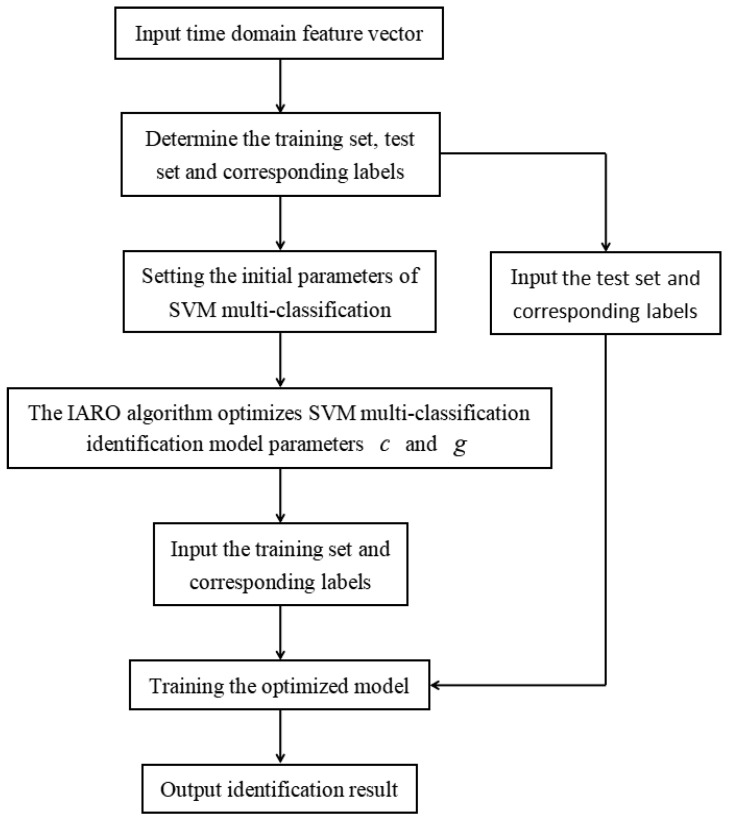
Identification model of vibration signals of hydraulic units based on IARO-SVM.

The identification of vibration states of hydraulic units is a multi-classification identification problem. The SVM [42,43] is a classification algorithm to solve binary classification problems, and it cannot be directly used to solve multi-classification problems. In this paper, the “one-to-one” (OVO) strategy is combined with SVM to form a multi-classifier. Therefore, the SVM multi-classifier design is applied to the identification of vibration signals in hydraulic units. After conducting VMD decomposition, the calculated time-domain feature vector is used as the input of the SVM multi-classifier. Combined with the IARO algorithm, the penalty parameter c and kernel function parameter g of the SVM multi-classifier are optimized. The IARO-SVM identification model is constructed, as shown in Figure 9, to identify the vibration states of the hydraulic units. 

### 5.5. Validation of the Proposed IARO-SVM Model

The parameters of the IARO-SVM model are initialized: the initial population number is 30, and the maximum number of iterations is 500. The time-domain feature vectors of each vibration state of the hydraulic unit are divided into the training set and the test set, in which the first 70% of data of each group of feature vectors are used as the training set, and the remaining 30% data are used as the test set. All data are normalized to form a sample set. After training the IARO-SVM model using the training samples, the test samples are input into the model.

**Figure 10:**
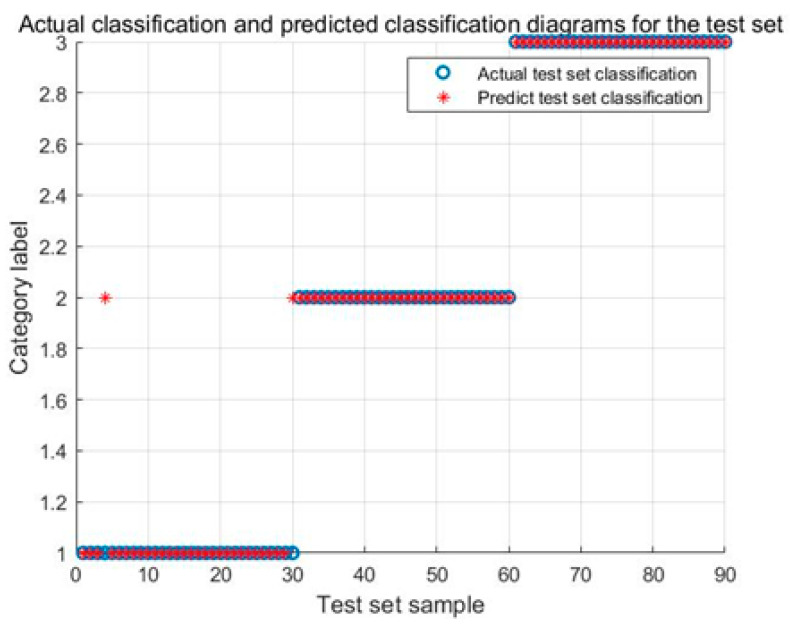
Identification results of the IARO-SVM model for test signals.

Figure 10 illustrates the identification results of the IARO-SVM model. The identification accuracy of the Karman vortex and the fault shutdown signals achieve 100%, and the identification accuracy of the power generation condition achieves 93.33%. 

To further verify the superiority of the IARO-SVM model, the results of the ARO-SVM model, ASO-SVM model, PSO-SVM model and WOA-SVM model are compared, respectively. 

**Figure 11:**
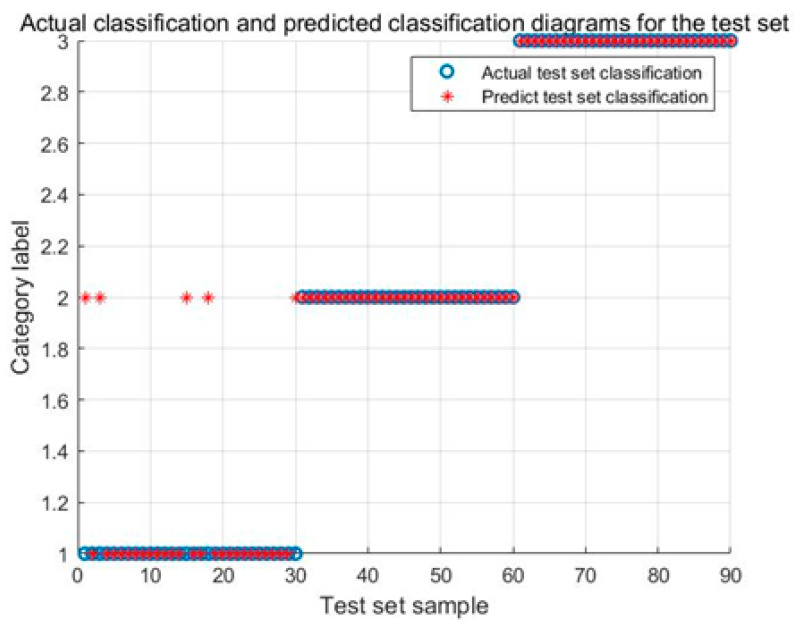
Identification results of the ARO-SVM model for test signals.

Figure 11 illustrates the identification results of the ARO-SVM model. The identification accuracy of the Karman vortex and the fault shutdown signals achieve 100%, and the identification accuracy of the power generation condition achieves 83.33%.

**Figure 12:**
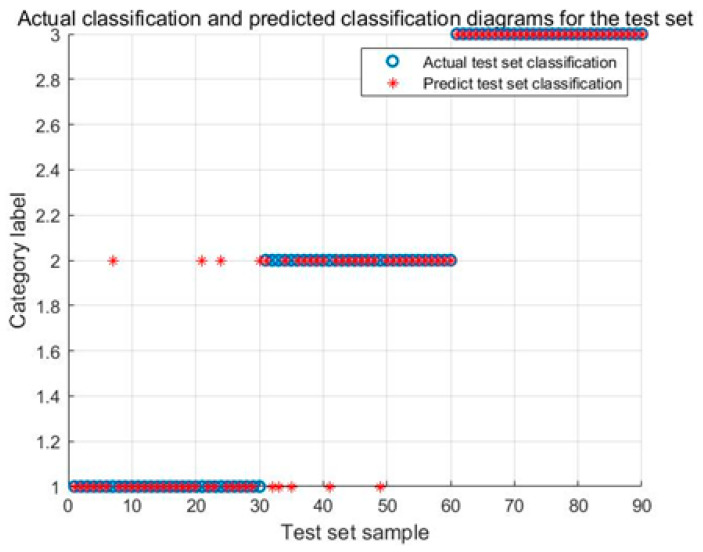
Identification results of the ASO-SVM model for test signals.

Figure 12 illustrates the identification results of the ASO-SVM model. The identification accuracy of the fault shutdown signals achieves 100%. The identification accuracy of the power generation condition and the Karman vortex achieve 86.67% and 83.33%, respectively.

**Figure 13:**
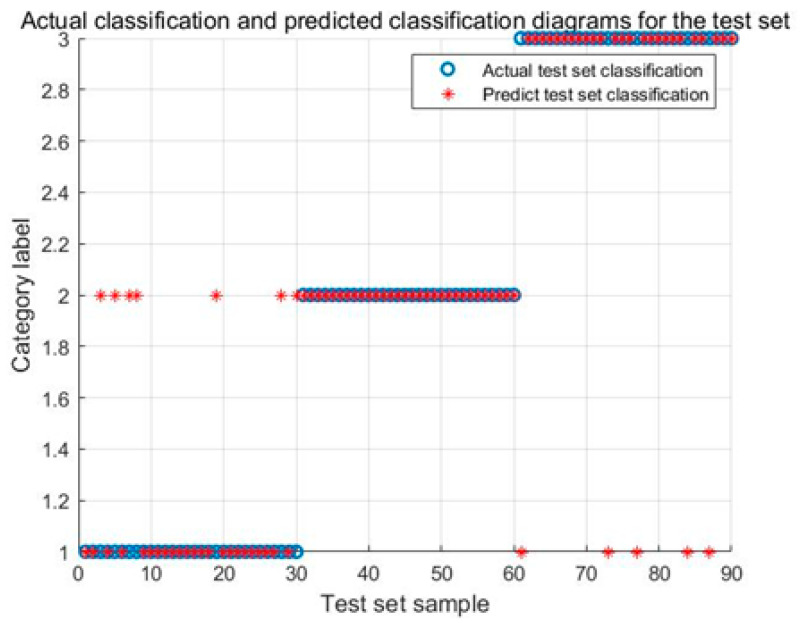
Identification results of the PSO-SVM model for test signals.

Figure 13 illustrates the identification results of the PSO-SVM model. The identification accuracy of the Karman vortex achieves 100%. The identification accuracy of the power generation condition and the fault shutdown achieve 76.67 and 83.33%, respectively.

**Figure 14:**
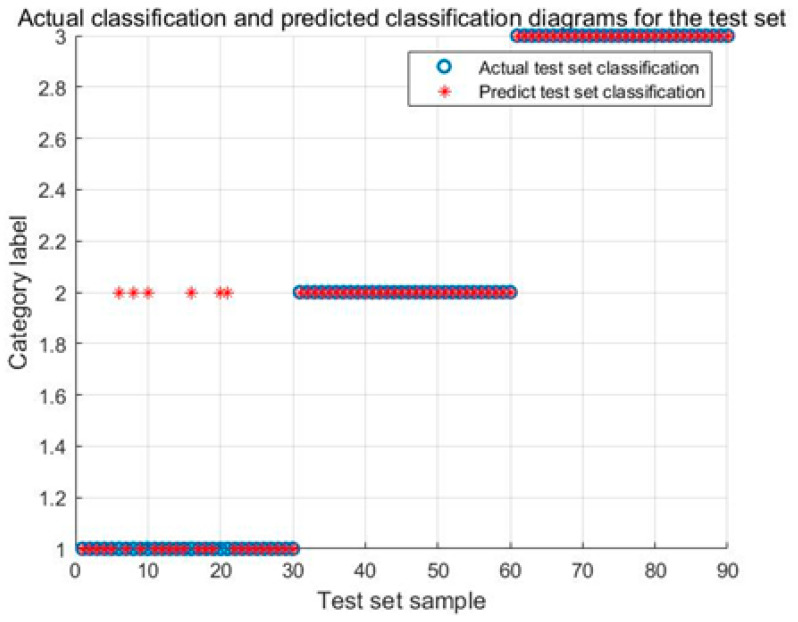
Identification results of the WOA-SVM model for test signals.

Figure 14 illustrates the identification results of the WOA-SVM model. The identification accuracy of the Karman vortex and the fault shutdown signals achieve 100%, and the identification accuracy of the power generation condition achieves 80.0%. Comparing Figure 10, Figure 11, Figure 12, Figure 13 and Figure 14, it can be found that the identification results about the power generation condition are obviously different and the IARO-SVM model achieves the best accuracy, while other models achieve poorer accuracies. This is due to that signal differentiation corresponding to the power generation condition is the lowest, reducing the identification accuracy of the models. However, the IARO-SVM model still keeps a higher identification accuracy, indicating the LARO algorithm has a better optimization performance for the SVM.

**Figure 15:**
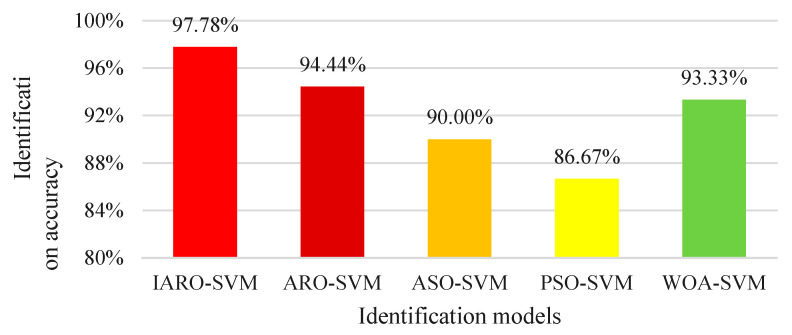
Average identification accuracy of different models.

After 3 experiments, the average identification accuracy of the IARO-SVM model, the ARO-SVM model, the ASO-SVM model, the PSO-SVM model and the WOA-SVM model are summarized in Figure 15. Based on Figure 15, the average identification accuracy of the IARO-SVM model is the best at 97.78%, which is followed by the ARO-SVM model (94.44%), WOA-SVM model (93.33%), ASO-SVM model (90.00%) and PSO-SVM model (86.67%). 

To further test the efficiency of the IARO-SVM model, the 2# Unit data is selected, and the same three vibration states need to be identified: the stability test of power generation condition, the Karman vortex street phenomenon test and the simulated fault shutdown test. Except for the different units selected, the methods of data collection and classification, processing and extraction, selection of feature vectors, training and identification are the same as those of the 1# Unit data.

**Figure 16:**
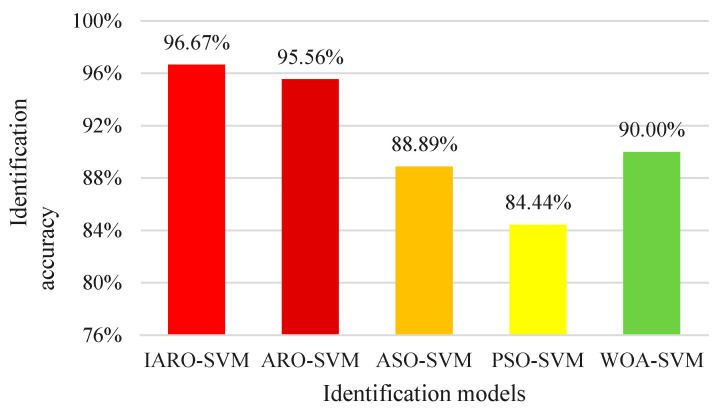
Average identification accuracy of different models.

The parameters of the IARO-SVM model, ARO-SVM model, ASO-SVM model, PSO-SVM model and WOA-SVM model are initialized in the same way: the initial population number is 30, and the maximum number of iterations is 500. The time-domain feature vectors of each vibration state are divided into the training set and test set, in which the first 70% of data of each group of feature vectors is used as a training set, and the remaining 30% of data is used as a test set. All data are normalized to form a sample set. After training and prediction, the average identification accuracy of the three vibration states of the 2# Unit is shown in Figure 16. Inspecting this figure, it is seen that, compared with other models, the IARO-SVM model obtains the best results with an average accuracy at 96.67%, followed by the ARO-SVM model (95.56%) and the WOA-SVM model (90.00%). 

## 6. Conclusions

This paper proposes an identification method based on ARO with the adaptive weight adjustment strategy to SVM (IARO-SVM). By introducing the adaptive weight adjustment strategy, the algorithm can adaptively change the weight size according to the distribution of the current rabbit population. Using 23 benchmark functions, IARO is compared with ARO, PSO, ASO and WOA, and the performance of IARO is better than that of other algorithms by comparing the minimum values of the four indicators and convergence curves.

This method optimizes the parameters of the SVM multi-classifier through the IARO algorithm. Meanwhile, the VMD method is used to decompose the vibration status signals of hydraulic units, and the time domain characteristic indicators of IMF components are extracted as multi-dimensional time-domain feature vectors and input into the IARO-SVM model, ARO-SVM model, ASO-SVM model, PSO-SVM model and WOA-SVM model for state identification of vibration signals. The experimental results show that the identification rate of the IARO-SVM model is 97.78%, the identification rate of the ARO-SVM model is 94.44%, the identification rate of the ASO-SVM model is 90.0%, the identification rate of PSO-SVM model is 86.67%, and the identification rate of WOA-SVM model is 93.33%. Through the comparative analysis of five models, the IARO-SVM model has obvious advantages over its competitors in the state identification of vibration signals of hydraulic units, which is 3.34% higher than the closest ARO-SVM model. For the second experiment, the IARO-SVM also achieve competitive results. Therefore, IARO has obvious advantages over other algorithms in optimizing the SVM for vibration status identification of hydraulic units.

Although the IARO-SVM model has excellent performance, it also has some limitations. (1) The amount of experimental data is limited, so in the experimental process, sometimes there will be large deviations, which will affect the accuracy of vibration state signal classification and identification. (2) Due to the experimental conditions, the data used in the paper is only the data of pumped storage hydraulic units, and the data including other types of hydraulic units cannot be verified, the state identification for other types needs further verification. However, these shortcomings are also directions for future research. On the one hand, the amount of data can be increased and the sample set capacity can be expanded when collecting data in the future. On the other hand, the vibration state signals of other types of hydraulic units are collected to verify whether the IARO-SVM model still has obvious advantages in vibration state classification and identification for other types.

## Figures and Tables

**Table 1:** Unimodal functions.

Name	Function	D	Range	$f_{opt}$
Sphere	$f_{1}(x)=\sum \begin{array}{l} n\\ i=1 \end{array}x_{i}^{2}$	30	${\left[-100,100\right]}^{D}$	0
Schwefel 2.22	$f_{2}(x)=\sum \begin{array}{l} n\\ i=1 \end{array}\left|x_{i}\right|+\prod_{i=1}^{n}\left|x_{i}\right|$	30	${\left[-10,10\right]}^{D}$	0
Schwefel 1.2	$f_{3}(x)=\sum \begin{array}{l} n\\ i=1 \end{array}(\sum_{j=1}^{i}x_{j}{)}^{2}$	30	${\left[-100,100\right]}^{D}$	0
Schwefel 2.21	$f_{4}(x)=\max_{i}\left\{\left|x_{i}\right|\right.,1\leq i\leq \left.n\right\}$	30	${\left[-100,100\right]}^{D}$	0
Rosenbrock	$f_{5}(x)=\sum_{i=1}^{n-1}(100(x_{i+1}-x_{i}{)}^{2}+{\left(x_{i}-1\right)}^{2})$	30	${\left[-30,30\right]}^{D}$	0
Step	$f_{6}(x)=\sum \begin{array}{l} n\\ i=1 \end{array}{\left(x_{i}+0.5\right)}^{2}$	30	${\left[-100,100\right]}^{D}$	0
Quartic	$f_{7}(x)=\sum \begin{array}{l} n\\ i=1 \end{array}ix_{i}^{4}+random\left[0,1\right)$	30	${\left[-1.28,1.28\right]}^{D}$	0

**Table 2:** Multimodal benchmark functions.

Name	Function	D	Range	$f_{opt}$
Schwefel	$f_{8}(x)=-\sum \begin{array}{l} n\\ i=1 \end{array}(x_{i}\sin(\sqrt{\left|x_{i}\right|}))$	30	${\left[-500,500\right]}^{D}$	−12,569.5
Rastrigin	$f_{9}(x)=\sum \begin{array}{l} n\\ i=1 \end{array}{\left(x_{i}^{2}-10\cos(2\pi x_{i})+10\right)}^{2}$	30	${\left[-5.12,5.12\right]}^{D}$	0
Ackley	$f_{10}(x)=-20\exp(-0.2\sqrt{\frac{1}{n}\sum \begin{array}{l} n\\ i=1 \end{array}x_{i}{}^{2}})-\exp(\frac{1}{n}\sum \begin{array}{l} n\\ i=1 \end{array}\cos2\pi x_{i})+20+e$	30	${\left[-32,32\right]}^{D}$	0
Griewank	$f_{11}(x)=\frac{1}{4000}\sum \begin{array}{l} n\\ i=1 \end{array}{\left(x_{i}-100\right)}^{2}-\prod_{i=1}^{n}\cos(\frac{x_{i}-100}{\sqrt{i}})+1$	30	${\left[-600,600\right]}^{D}$	0
Penalized	$\begin{array}{l} f_{12}(x)=\frac{\pi }{n}\left\{10\sin^{2}(\pi y_{1})\right.+\sum_{i=1}^{n-1}(y_{i}-1{)}^{2}\left[1+10\sin^{2}(\pi y_{i}+1)\right]\\ +\left.{\left(y_{n}-1\right)}^{2}\right\}+\sum_{i=1}^{30}u(x_{i},10,100,4) \end{array}$	30	${\left[-50,50\right]}^{D}$	0
Penalized 2	$\begin{array}{l} f_{13}(x)=0.1\left\{\sin^{2}(3\pi x_{1})\right.+\sum_{i=1}^{29}(x_{i}-1{)}^{2}p\left[1+\sin^{2}(3\pi x_{i+1})\right]\\ +\left.{\left(x_{n}-1\right)}^{2}\left[1+\sin^{2}(2\pi x_{30})\right]\right\}+\sum_{i=1}^{30}u(x_{i},5,10,4) \end{array}$	30	${\left[-50,50\right]}^{D}$	0

**Table 3:** Fixed dimension multimodal functions.

Name	Function	D	Range	$f_{opt}$
Foxholes	$f_{14}\left(x\right)={\left[\frac{1}{500}+\sum_{j=1}^{25}\frac{1}{j+\sum_{j=1}^{2}{\left(x_{i}-a_{ij}\right)}^{6}}\right]}^{-1}$	2	${\left[-65.536,65.536\right]}^{D}$	0.998
Kowalik	$f_{15}(x)={\sum_{i=1}^{11}\left|a_{i}-\frac{x_{1}(b_{i}^{2}+b_{i}x_{2})}{b_{i}^{2}+b_{i}x_{3}+x_{4}}\right|}^{2}$	4	${\left[-5,5\right]}^{D}$	$3.075\times 10^{-4}$
Six Hump Camel	$f_{16}(x)=4x_{1}^{2}-2.1x_{1}^{4}+\frac{1}{3}x_{1}^{6}+x_{1}x_{2}-4x_{2}^{2}+4x_{2}^{4}$	2	${\left[-5,5\right]}^{D}$	−1.0316
Branin	$f_{17}(x)={\left(x_{2}-\frac{5.1}{4\pi^{2}}x_{1}^{2}+\frac{5}{\pi }x_{1}-6\right)}^{2}+10(1-\frac{1}{8\pi })\cos x_{1}+10$	2	$\left[-5,10\right]\times \left[0,15\right]$	0.398
Goldstein-Price	$\begin{array}{l} f_{18}(x)=\left[1+{\left(x_{1}+x_{2}+1\right)}^{2}(19-14x_{1}+3x_{1}^{2}-14x_{2}\right.\\ +6x_{1}x_{2}+\left.3x_{2}^{2})\right]\times \left[30+(2x_{1}\right.+1-3x_{2}{)}^{2}(18-32x_{1}\\ +12x_{1}^{2}+48x_{2}-36x_{1}x_{2}+\left.27x_{2}^{2})\right] \end{array}$	2	${\left[-2,2\right]}^{D}$	3
Hartman 3	$f_{19}(x)=-\sum_{i=1}^{4}\exp\left[-\sum_{j=1}^{3}a_{ij}{\left(x_{j}-p_{ij}\right)}^{2}\right]$	3	${\left[0,1\right]}^{D}$	−3.86
Hartman 6	$f_{20}(x)=-\sum_{i=1}^{4}\exp\left[-\sum_{j=1}^{6}a_{ij}{\left(x_{j}-p_{ij}\right)}^{2}\right]$	6	${\left[0,1\right]}^{D}$	−3.322
Shekel 5	$f_{21}(x)=-{\sum_{i=1}^{5}\left|\left(x_{i}-a_{i}\right){\left(x_{i}-a_{i}\right)}^{T}+c_{i}\right|}^{-1}$	4	${\left[0,10\right]}^{D}$	−10.1532
Shekel 7	$f_{22}(x)=-{\sum_{i=1}^{7}\left|\left(x_{i}-a_{i}\right){\left(x_{i}-a_{i}\right)}^{T}+c_{i}\right|}^{-1}$	4	${\left[0,10\right]}^{D}$	−10.4028
Shekel 10	$f_{23}(x)=-{\sum_{i=1}^{10}\left|\left(x_{i}-a_{i}\right){\left(x_{i}-a_{i}\right)}^{T}+c_{i}\right|}^{-1}$	4	${\left[0,10\right]}^{D}$	−10.5363

**Table 4:** Algorithm results of the unimodal test function.

NO.	INDEX	IARO	ARO	PSO	ASO	WOA
F1	Mean	**0**	1.89 × 10^−34^	9.08 × 10^−50^	1.47 × 10^−33^	2.31 × 10^−16^
	Std	**0**	6.59 × 10^−34^	4.78 × 10^−49^	7.15 × 10^−33^	2.68 × 10^−16^
	Worst	**0**	3.07 × 10^−33^	2.62 × 10^−48^	3.93 × 10^−32^	1.03 × 10^−15^
	Best	**0**	1.89 × 10^−34^	9.08 × 10^−50^	1.47 × 10^−33^	2.31 × 10^−16^
F2	Mean	**0**	1.04 × 10^−19^	1.09 × 10^−32^	1.56 × 10^−19^	3.12 × 10^−4^
	Std	**0**	2.18 × 10^−19^	3.06 × 10^−32^	3.24 × 10^−19^	1.02 × 10^−3^
	Worst	**0**	8.70 × 10^−19^	1.61 × 10^−31^	1.12 × 10^−18^	4.18 × 10^−3^
	Best	**0**	1.04 × 10^−19^	1.09 × 10^−32^	1.56 × 10^−19^	3.12 × 10^−4^
F3	Mean	**0**	4.82 × 10^−25^	4.98 × 10^+4^	1.52 × 10^−22^	3.13 × 10^+3^
	Std	**0**	1.45 × 10^−24^	1.54 × 10^+4^	8.21 × 10^−22^	9.39 × 10^+2^
	Worst	**0**	7.70 × 10^−24^	8.59 × 10^+4^	4.50 × 10^−21^	5.16 × 10^+3^
	Best	**0**	4.82 × 10^−25^	4.98 × 10^+4^	1.52 × 10^−22^	3.13 × 10^+3^
F4	Mean	**0**	1.78 × 10^−14^	4.35 × 10^+1^	5.01 × 10^−14^	8.70 × 10^−2^
	Std	**0**	5.07 × 10^−14^	3.20 × 10^+1^	2.16 × 10^−13^	1.68 × 10^−1^
	Worst	**0**	2.14 × 10^−13^	8.788 × 10^+1^	1.18 × 10^−12^	7.08 × 10^−1^
	Best	**0**	1.78 × 10^−14^	4.35 × 10^+1^	5.01 × 10^−14^	8.70 × 10^−2^
F5	Mean	1.55 × 10^+0^	2.01 × 10^+0^	2.80 × 10^+1^	**7.43 × 10^−1^**	6.16 × 10^+1^
	Std	3.16 × 10^+0^	5.10 × 10^+0^	**3.34 × 10^−1^**	9.57 × 10^−1^	9.49 × 10^+1^
	Worst	1.50 × 10^+1^	2.69 × 10^+1^	2.87 × 10^+1^	**4.68 × 10^+0^**	5.31 × 10^+2^
	Best	1.55 × 10^+0^	2.01 × 10^+0^	2.80 × 10^+1^	**7.43 × 10^−1^**	6.16 × 10^+1^
F6	Mean	**0**	**0**	**0**	**0**	4.0 × 10^−1^
	Std	**0**	**0**	**0**	**0**	8.14 × 10^−1^
	Worst	**0**	**0**	**0**	**0**	4
	Best	**0**	**0**	**0**	**0**	4.0 × 10^−1^
F7	Mean	**8.92 × 10^−5^**	9.02 × 10^−4^	4.04 × 10^−3^	9.43 × 10^−4^	5.78 × 10^−2^
	Std	**9.76 × 10^−5^**	5.93 × 10^−4^	3.76 × 10^−3^	6.21 × 10^−4^	2.14 × 10^−2^
	Worst	**4.35 × 10^−4^**	2.37 × 10^−3^	1.50 × 10^−2^	2.59 × 10^−3^	1.17 × 10^−1^
	Best	**8.92 × 10^−5^**	9.02 × 10^−4^	4.04 × 10^−3^	9.43 × 10^−4^	5.78 × 10^−2^

**Table 5:** Results of algorithms of the multimodal functions.

NO.	INDEX	IARO	ARO	PSO	ASO	WOA
F8	Mean	−9.90 × 10^+3^	**−1.02 × 10^+4^**	**−1.02 × 10^+4^**	**−1.02 × 10^+4^**	−7.18 × 10^+3^
	Std	**4.68 × 10^+2^**	5.09 × 10^+2^	1.73 × 10^+3^	5.68 × 10^+2^	6.89 × 10^+2^
	Worst	**−9.03 × 10^+3^**	−8.79 × 10^+3^	−7.79 × 10^+3^	−8.98 × 10^+3^	−5.88 × 10^+3^
	Best	−9.90 × 10^+3^	**−1.02 × 10^+4^**	**−1.02 × 10^+4^**	**−1.02 × 10^+4^**	−7.18 × 10^+3^
F9	Mean	**0**	**0**	**0**	**0**	3.09 × 10^+1^
	Std	**0**	**0**	**0**	**0**	9.33
	Worst	**0**	**0**	**0**	**0**	4.78 × 10^+1^
	Best	**0**	**0**	**0**	**0**	3.09 × 10^+1^
F10	Mean	**8.88 × 10^−16^**	**8.88 × 10^−16^**	5.27 × 10^−15^	1.01 × 10^−15^	7.98 × 10^−9^
	Std	**0**	**0**	2.22 × 10^−15^	6.49 × 10^−16^	4.43 × 10^−9^
	Worst	**8.88 × 10^−16^**	**8.88 × 10^−16^**	7.99 × 10^−15^	4.44 × 10^−15^	2.36 × 10^−8^
	Best	**8.88 × 10^−16^**	**8.88 × 10^−16^**	5.27 × 10^−15^	1.01 × 10^−15^	7.98 × 10^−9^
F11	Mean	**0**	**0**	4.86 × 10^−3^	**0.00 × 10^+0^**	1.31 × 10^−3^
	Std	**0**	**0**	2.66 × 10^−2^	**0**	4.51 × 10^−3^
	Worst	**0**	**0**	1.46 × 10^−1^	**0**	2.21 × 10^−2^
	Best	**0**	**0**	4.86 × 10^−3^	**0**	1.31 × 10^−3^
F12	Mean	5.22 × 10^−3^	**4.51 × 10^−4^**	1.62 × 10^−2^	5.13 × 10^−4^	2.11 × 10^−2^
	Std	3.73 × 10^−3^	4.31 × 10^−4^	9.48 × 10^−3^	**3.14 × 10^−4^**	4.29 × 10^−2^
	Worst	1.68 × 10^−2^	2.44 × 10^−3^	3.78 × 10^−2^	**1.62 × 10^−3^**	1.30 × 10^−1^
	Best	5.22 × 10^−3^	**4.51 × 10^−4^**	1.62 × 10^−2^	5.13 × 10^−4^	2.11 × 10^−2^
F13	Mean	4.61 × 10^−2^	8.14 × 10^−3^	4.26 × 10^−1^	**6.03 × 10^−3^**	7.33 × 10^−3^
	Std	3.64 × 10^−2^	1.09 × 10^−2^	2.05 × 10^−1^	**9.97 × 10^−3^**	1.94 × 10^−2^
	Worst	1.37 × 10^−1^	**4.47 × 10^−2^**	8.31 × 10^−1^	4.59 × 10^−2^	9.89 × 10^−2^
	Best	4.61 × 10^−2^	8.14 × 10^−3^	4.26 × 10^−1^	**6.03 × 10^−3^**	7.33 × 10^−3^

**Table 6:** Algorithm results of the fixed dimension multimodal test functions.

NO.	INDEX	IARO	ARO	PSO	ASO	WOA
F14	Mean	**9.98 × 10^−1^**	**9.98 × 10^−1^**	1.8859	**9.98 × 10^−1^**	1.5523
	Std	**7.14 × 10^−17^**	**7.14 × 10^−17^**	1.88	8.25 × 10^−17^	6.76 × 10^−1^
	Worst	**9.98 × 10^−1^**	**9.98 × 10^−1^**	10.76	**9.98 × 10^−1^**	3.1209
	Best	**9.98 × 10^−1^**	**9.98 × 10^−1^**	1.8859	**9.98 × 10^−1^**	1.5523
F15	Mean	3.76 × 10^−4^	3.27 × 10^−4^	6.53 × 10^−4^	**3.12 × 10^−4^**	1.31 × 10^−3^
	Std	8.24 × 10^−5^	3.56 × 10^−5^	4.16 × 10^−4^	**7.47 × 10^−6^**	1.02 × 10^−3^
	Worst	6.44 × 10^−4^	4.59 × 10^−4^	2.25 × 10^−3^	**3.37 × 10^−4^**	6.49 × 10^−3^
	Best	3.76 × 10^−4^	3.27 × 10^−4^	6.53 × 10^−4^	**3.12 × 10^−4^**	1.31 × 10^−3^
F16	Mean	**−1.0316**	**−1.0316**	**−1.0316**	**−1.0316**	**−1.0316**
	Std	**5.17 × 10^−16^**	5.56 × 10^−16^	1.93 × 10^−9^	5.30 × 10^−16^	5.98 × 10^−16^
	Worst	**−1.0316**	**−1.0316**	**−1.0316**	**−1.0316**	**−1.0316**
	Best	**−1.0316**	**−1.0316**	**−1.0316**	**−1.0316**	**−1.0316**
F17	Mean	**3.98 × 10^−1^**	**3.98 × 10^−1^**	**3.98 × 10^−1^**	**3.98 × 10^−1^**	**3.98 × 10^−1^**
	Std	0	**6.97 × 10^−14^**	7.68 × 10^−6^	8.21 × 10^−14^	0
	Worst	**3.98 × 10^−1^**	**0.3979**	**3.98 × 10^−1^**	**3.98 × 10^−1^**	**3.98 × 10^−1^**
	Best	**3.98 × 10^−1^**	**3.98 × 10^−1^**	**3.98 × 10^−1^**	**3.98 × 10^−1^**	**3.98 × 10^−1^**
F18	Mean	**3.0000**	**3.0000**	3.9003	**3.0000**	**3.0000**
	Std	1.50 × 10^−15^	**5.34 × 10^−16^**	4.93 × 10^−1^	1.34 × 10^−15^	1.61 × 10^−15^
	Worst	**3.0000**	**3.0000**	30.0077	**3.0000**	**3.0000**
	Best	**3.0000**	**3.0000**	3.9003	**3.0000**	**3.0000**
F19	Mean	**−3.8628**	**−3.8628**	−3.8576	**−3.8628**	**−3.8628**
	Std	2.52 × 10^−15^	2.49 × 10^−15^	9.09 × 10^−3^	**2.46 × 10^−15^**	2.60 × 10^−15^
	Worst	**−3.8628**	**−3.8628**	−3.8235	**−3.8628**	**−3.8628**
	Best	**−3.8628**	**−3.8628**	−3.8576	**−3.8628**	**−3.8628**
F20	Mean	−3.2943	−3.3101	−3.2848	−3.3141	**−3.3220**
	Std	5.11 × 10^−2^	3.63 × 10^−2^	6.57 × 10^−2^	3.02 × 10^−2^	**1.36 × 10^−15^**
	Worst	−3.2031	−3.2031	−3.0778	−3.2031	**−3.3220**
	Best	−3.2943	−3.3101	−3.2848	−3.3141	**−3.3220**
F21	Mean	−9.9787	**−10.1531**	−7.6137	−10.1530	−8.47
	Std	8.37 × 10^−1^	**8.12 × 10^−4^**	3.0335	1.27 × 10^−3^	3.1122
	Worst	−5.5962	**−10.1488**	−2.6296	−10.1463	−2.6829
	Best	−9.9787	**−10.1531**	−7.6137	−10.1530	−8.4749
F22	Mean	**−10.4029**	−10.2258	−7.8582	−10.1794	−9.8929
	Std	**1.38 × 10^−10^**	9.70 × 10^−1^	2.86	1.22	1.942
	Worst	**−10.4029**	−5.0877	−2.7642	−3.7243	−2.7519
	Best	**−10.4029**	−10.2258	−7.8582	−10.1794	−9.8929
F23	Mean	**−10.5364**	**−10.5364**	−7.8230	**−10.5364**	**−10.5364**
	Std	6.00 × 10^−5^	2.54 × 10^−7^	3.1920	2.03 × 10^−11^	**1.32 × 10^−15^**
	Worst	−10.5361	**−10.5364**	−2.4216	**−10.5364**	**−10.5364**
	Best	**−10.5364**	**−10.5364**	−7.8230	**−10.5364**	**−10.5364**

**Table 7:** Classification of experimental data.

Vibration State	Measuring Point Position	Experimental Point	Direction	Category Label
Stability test of power generation condition	upper guide bearing	1	X	1
	upper guide bearing	2	Y	
	water guide bearing	3	X	
	water guide bearing	4	Y	
	lower guide bearing	5	X	
	lower guide bearing	6	Y	
Karman vortex street phenomenon test	upper guide bearing	1	X	2
	upper guide bearing	2	Y	
	water guide bearing	3	X	
	water guide bearing	4	Y	
	lower guide bearing	5	X	
	lower guide bearing	6	Y	
Simulated fault shutdown test	upper guide bearing	1	X	3
	upper guide bearing	2	Y	
	water guide bearing	3	X	
	water guide bearing	4	Y	
	lower guide bearing	5	X	
	lower guide bearing	6	Y	

**Table 8:** Number of decomposition layers and update steps of different vibration signals.

Vibration State	Measuring Point Position and Direction	Number of Decomposition Layers (K)	Update Steps (tau)
Stability test of power generation condition	upper guide bearing, X direction	13	0.0043
	upper guide bearing, Y direction	12	0.0312
	water guide bearing, X direction	13	0.0012
	water guide bearing, Y direction	12	0.0315
	lower guide bearing, X direction	12	0.0314
	lower guide bearing, Y direction	12	0.0068
Karman vortex street phenomenon test	upper guide bearing, X direction	14	0.0192
	upper guide bearing, Y direction	13	0.0321
	water guide bearing, X direction	12	0.0077
	water guide bearing, Y direction	12	0.1331
	lower guide bearing, X direction	15	0.0205
	lower guide bearing, Y direction	11	0.0474
Simulated fault shutdown test	upper guide bearing, X direction	12	0.0318
	upper guide bearing, Y direction	15	0.0483
	water guide bearing, X direction	10	0.3245
	water guide bearing, Y direction	12	0.0505
	lower guide bearing, X direction	14	0.0230
	lower guide bearing, Y direction	12	0.0053

## Data Availability

Not applicable.
